# A Systematic Approach to Bacterial Phylogeny Using Order Level Sampling and Identification of HGT Using Network Science

**DOI:** 10.3390/microorganisms8020312

**Published:** 2020-02-24

**Authors:** Ehdieh Khaledian, Kelly A. Brayton, Shira L. Broschat

**Affiliations:** 1 School of Electrical Engineering and Computer Science, Washington State University, P.O. Box 642752, Pullman, WA 99164, USA; kbrayton@wsu.edu (K.A.B.); shira@wsu.edu (S.L.B.); 2 Department of Veterinary Microbiology and Pathology, Washington State University, P.O. Box 647040, Pullman, WA 99164, USA; 3 Paul G. Allen School for Global Animal Health, Washington State University, P.O. Box 647090, Pullman, WA 99164, USA

**Keywords:** tree of bacterial phyla, phylogeny, network science, network of bacteria, horizontal gene transfer

## Abstract

Reconstructing and visualizing phylogenetic relationships among living organisms is a fundamental challenge because not all organisms share the same genes. As a result, the first phylogenetic visualizations employed a single gene, e.g., rRNA genes, sufficiently conserved to be present in all organisms but divergent enough to provide discrimination between groups. As more genome data became available, researchers began concatenating different combinations of genes or proteins to construct phylogenetic trees believed to be more robust because they incorporated more information. However, the genes or proteins chosen were based on ad hoc approaches. The large number of complete genome sequences available today allows the use of whole genomes to analyze relationships among organisms rather than using an ad hoc set of genes. We present a systematic approach for constructing a phylogenetic tree based on simultaneously clustering the complete proteomes of 360 bacterial species. From the homologous clusters, we identify 49 protein sequences shared by 99% of the organisms to build a tree. Of the 49 sequences, 47 have homologous sequences in both archaea and eukarya. The clusters are also used to create a network from which bacterial species with horizontally-transferred genes from other phyla are identified.

## 1. Introduction

New gene sequencing technologies have created an enormous increase in the number of complete genome sequences available to the public. This availability has presented scientists with an unprecedented opportunity to mine the knowledge they contain. One of our strategies has been to use a fast and accurate software tool, *pClust* [1], to group protein sequences into homologous clusters. In this paper, we show that such clusters can be used to construct a phylogenetic tree in a systematic manner using proteins shared by 99% or more of all the organisms. Our protein clusters can also be used to create a bacterial network, and network science, the study of complex networks based on graph theory and other mathematical fields [2], can be used to analyze this network to understand the relationships between the recognized bacterial phyla. In addition, this network can be used to identify bacterial species with numerous horizontally-transferred genes. Identifying such genes is important as they can introduce radically different genotypes from distant lineages or new functions, and, as such, they can be a major source of phenotypic innovation. For example, of particular relevance to human health is the lateral transfer of antibiotic resistance and pathogenicity determinants, leading to the emergence of pathogenic lineages [3]. In recent years, the concept of the rhizome or network of life, which incorporates horizontal gene transfer (HGT) and a more complex view of relationships between organisms, has been proposed as an alternative to the phylogenetic tree of life [4].

In this study we developed a systematic phylogenomics analysis pipeline by leveraging publicly available genomic data. We determined that in order to study the interphylum relationships of bacteria, it is necessary to select genomes at the order level to capture pertinent diversity and to avoid bias in the dataset. This is because of the non-uninform distribution of genomes across phyla currently available at the National Center for Biotechnology Information (NCBI). For example, a single phylum, Proteobacteria, represents approximately half of all the genomes in the NCBI database. Originally, we randomly sampled from the genomes available in different phyla, but this led to oversampling of Proteobacteria and, in particular, Gammaproteobacteria which account for about half of all Proteobacterial genomes. Thus, we rethought our approach, examining the number of different orders within classes, and concluded that sampling at the order level was optimum. To analyze the complete genomes from a set of bacterial organisms we first clustered every protein sequence deduced from the genomes. We clustered representatives from all but two recognized bacterial phyla [5] comprising 194 orders [6,7] and applied network science techniques to visualize and interpret the results. Our work is distinct from other computational approaches in that all protein sequences for the 360 organisms used in the study were clustered concurrently using *pClust*. From the 360 genomes, we were able to identify a set of 122 essential genes shared by more than 94% of the organisms across all phyla and a smaller set of 49 shared by 99% or more of the bacteria in the study. Of these 49 genes, 47 are present in representatives of both archaea and eukarya. Protein sequences for the 49 genes were used to create phylogenetic trees which were compared with other recently generated trees [8,9]. In addition, from the network of bacterial organisms created from the clusters we were able to discern genomes with extensive HGT. We then identified the individual horizontally-transferred genes using an established approach [10].

## 2. Materials and Methods

### 2.1. Datasets and Clustering

Proteomes deduced from complete bacterial genomes were downloaded from the NCBI database [7]. We determined that to avoid biasing the data due to the predominance of specific phyla and to capture sufficient diversity in our dataset, genomes needed to be sampled by orders rather than by phyla or by random selection. A list of orders and their availability at NCBI is given in Supplementary Table S1. We downloaded up to three organisms per order (if available) for each dataset. Organisms for the three different datasets (Dataset 1, Dataset 2, and Dataset 3) used are listed in Supplementary Tables S2–S4 with their accession numbers. The total number of protein sequences in each of the datasets varied with the largest containing approximately 1.2 M. The *pClust* pipeline was employed to group the protein sequences into homologous clusters. *pClust* is an open source software package that uses the highly efficient software package Parasail [11] for sequence alignment and Grappolo [12] for clustering to achieve fast and efficient clustering of protein sequences. Parasail employs a filtering approach to reduce the number of pair-wise sequence alignments to approximately 0.1% of the original number and allows the user to choose between local, semi-global, and global alignment. Semi-global alignment was used for our work. Grappolo applies the Louvain community detection algorithm to the graph results from Parasail to detect clusters. The Louvain community detection algorithm finds clusters by optimizing a modularity metric [13]. We used default settings for *pClust* on a desktop computer running Windows 7 with 128 GB of RAM and 12 cores. The run-time for the largest dataset was 54 h. The output of *pClust* is a text file listing the cluster number, the number of protein sequences in each cluster, and identification of the protein sequences in each cluster. Clusters with only one protein sequence or with more than one sequence but from the same organism, called singletons, were not used in this study. Our 360-organism dataset resulted in 110,495 non-singleton clusters, i.e., clusters with a minimum of two protein sequences from more than one organism. The cluster results for the three datasets are available in [14].

### 2.2. Heatmap and Tree of Bacterial Phyla

For each dataset, we used the cluster results to create an n∗m matrix with *n* rows representing the number of protein clusters and *m* columns representing the number of organisms. Entries in the matrix consisted of 0′s and 1′s, with 0 indicating the absence of a protein sequence in an organism and 1 indicating the presence. Using the *R* igraph package [15] and the zero-one matrix we performed hierarchical clustering of the organisms, measuring the dissimilarity of observations via the Manhattan distance (see Section 2.3) and Ward’s minimum variance method. The latter minimizes the total within-cluster variance where cluster here refers to a cluster created during the hierarchical clustering procedure and is not to be confused with a protein cluster [16]. With Ward’s minimum variance method, the pairs of clusters with minimum between-cluster distance are merged at each step. Because the majority of protein sequence clusters were small and contained sequences from very few different organisms, we used only those clusters consisting of sequences from more than 10% of the organisms to reduce the computational load. This resulted in the use of approximately 4500 of the original 110,495 non-singleton clusters for the 360-organism dataset with an average of 120 different organisms per cluster.

**Figure 1:**
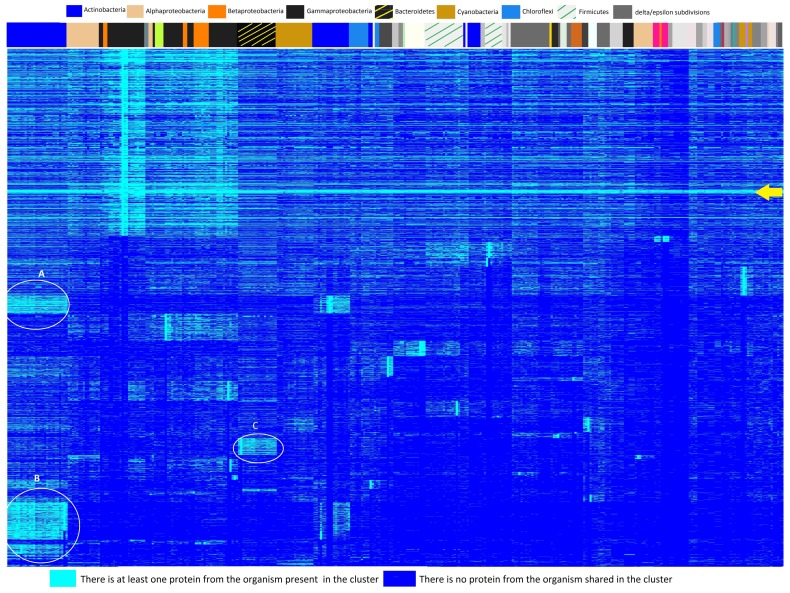
A heatmap of protein sequence cluster membership together with hierarchical clustering results for the 360 organisms. The heatmap assists in understanding the cluster results. Each row indicates a cluster, and each column represents an organism. Cyan represents membership in a protein cluster while blue indicates absence. The legend of phyla at the top of the figure shows the largest clades represented by colored bands immediately above the heatmap. On the left side of the dendrogram we find Actinobacteria, with distinct blocks of clusters indicated by cyan (A and B in the figure). Bacteroidetes (black with yellow lines) also has blocks of protein clusters (C in the figure). The yellow arrow indicates the band of protein clusters broadly conserved across the phyla.

To determine which clusters contained proteins from ≥99% and >94% of organisms we wrote a C++ program which summed the number of ones in the zero-one matrix for each cluster. We considered clusters with ≥99% organisms to determine the most highly conserved proteins and clusters with >94% because they represent the cyan bar in Figure 1. A list of the 49 protein sequences shared by ≥99% of organisms is available in Supplementary Table S5, and a list of the 122 protein sequences shared by >94% of organisms is available in Supplementary Table S6.

We created a tree of bacterial phyla using the 49 highly-conserved proteins shared by ≥99% of the organisms in Dataset 1. To create the tree, multiple sequence alignments of the protein sequences corresponding to the 49 proteins were performed using MAFFT [17] which was chosen because of its accuracy, important for tree construction [18,19]. After concatenating the multiple-sequence alignment results, we used IQTree [20] to compute phylogenies using the maximum likelihood criterion which compares favorably to RAxML and PhyML which have similar compute times [20]. Finally we visualized the tree using ITOLS [21].

### 2.3. Network of Organisms

We computed the Manhattan distance between proteins in the different organisms as given by:

(1) $$d\left(p,q\right)=||p-q||=\sum_{i=1}^{n}p_{i}-q_{i}$$

where *p* and *q* are vectors $p=(p_{1},p_{2},\ldots ,p_{n})$, $q=(q_{1},q_{2},\ldots ,q_{n})$, and *n* is the number of clusters. The Manhattan distance, also called the $L_{1}$ or taxicab distance, basically measures the number of changes from 1 to 0 or vice versa to transform from one organism to another [22,23]. The resulting m∗m distance matrix describing the relationships between the organisms was normalized by dividing each entry by its row sum and taking the reciprocal of the result, and this matrix, the adjacency matrix, was used as the input for our network visualization. An adjacency matrix is a square matrix that shows the relationships between the nodes and edges in a network. We applied the maximum degree centrality measure [2] to the network of organisms to identify the most central organism. The central organism here is the organism that shares the highest number of proteins with other organisms from different phyla. We added the most central organism from each dataset to the other datasets. The R package igraph [24] was used to compute the centralities. To visualize the network of organisms the adjacency matrix was used with *visone* 2.16 [25], a powerful tool for visualizing and analyzing networks. We applied a weight threshold to remove extraneous low-weight edges to sparsify the network. Sparsification results in a network with fewer edges and nodes while maintaining meaningful structure. We applied 80% sparsification using the backbone layout of *visone*. This layout greatly reduces the number of weak edges while maintaining the connectedness of the network. Next, we extracted the adjacency matrix of the sparsified network using the *R* console in *visone* for our network analysis. Finally, we applied 70% quadrilateral sparsification to provide visual clarification of the network. Quadrilateral sparsification is based on a spanning subgraph that is sparse but connected and consists of strong ties holding communities together [26].

### 2.4. Identification of Horizontally-Transferred Genes and Protein Functions

In our bacterial network, an organism may contain an extensive number of genes horizontally transferred from other phyla when its closest neighbors in the network are phyla other than the one to which it belongs. To identify such horizontally-transferred genes, we first computed the G + C content of the first, second, and third codon positions and the total G+C content, referred to as GC1, GC2, GC3, and GCT, respectively. We used the GC function in the *R*-seqinr [27] package to calculate the G + C content for all sequences. Next, a gene was passed to the next step when GCT>μ+1.5δ or GCT<μ−1.5δ or when both GC1>μ+1.5δ and GC3>μ+1.5δ, where δ is the standard deviation of the G+C content and μ is the mean calculated using sequences with length greater than 300 bp. Genes passing this step were searched against the NCBI NR database using BLASTp [28]. We excluded genes of the same genus from the search. For example, we removed the *Mesotoga* species when searching for genes belonging to *Mesotoga infera* (*M. infera*). If the top ten hits in the search did not belong to the same phylum (Thermotogae) as *M. infera*, we identified them as candidates acquired by HGT.

For HGT candidates with unknown function, we investigated the nearest neighbors obtained from the BLASTp search. We considered the function of a gene to be the same as its neighbors. We then used Gene Ontology (GO [29]) to validate the results.

## 3. Results

### 3.1. Clustering Protein Sequences

At the initiation of this study, there were approximately 15,000 complete genomes available in the NCBI database for 33 of the 35 recognized bacterial phyla [5]. Two phyla had no species representation. Ideally, species in the database would have equal representation across all phyla, but such is not the case. Approximately half of the 15,000 genomes available were for one phylum, Proteobacteria. Therefore, if we were to select the organisms for our study using random sampling, or based on phyla, our dataset would be biased because of the overrepresentation of organisms in some phyla, e.g., *Escherichia coli* in the phylum Proteobacteria or *Bacillus* in the phylum Firmicutes. We determined that to avoid biasing the data due to the predominance of specific phyla and to capture sufficient diversity in our dataset, genomes needed to be sampled by orders rather than by phyla or by random selection. However, the beneficial impact of dense taxonomic sampling on phylogeny estimation was established many decades ago [30], and it has been emphasized in modern phylogenomic estimation [31]. Increased taxon sampling may improve the estimation of molecular rates and variation in base composition and thus result in improvements in estimates of tree topology [31]. As such, we compromised by selecting three complete genomes per order when available in NCBI and one or two otherwise.

In addition, we considered the classes of Proteobacteria rather than the phylum as a whole because Proteobacteria are not monophyletic [8], that is, they have not descended from a common evolutionary ancestor. Also, the phyla Bacteroidetes and Chlorobi are combined as a group at NCBI. Thus, our analysis is presented in terms of 37 groups that we will refer to as “phyla” for simplicity, representing 30 phyla, one combined group of two phyla, and seven classes that compose one phylum. There are 194 orders in the 37 phyla [6,7], but complete genomes were not available at NCBI for 50 of these orders at the time of this study. Moreover, only one complete genome was available for 28 of the 194 orders and two complete genomes for 15. A list of orders and their availability at NCBI is given in Supplementary Table S1.

Our goal was to create a phylogenetic network representing diverse organisms chosen from the 146 of 194 available orders. Dataset 1 was the only dataset used for our HGT study, but otherwise we performed our analyses in triplicate to ensure consistency of results (Dataset 1, Dataset 2, and Dataset 3). We assembled the three datasets with genomes from 358 organisms, 111 common to each dataset (due to a lack of other genomes in certain orders) and 247 unique to each dataset. We then determined the most central organism in each dataset (here, the one sharing the greatest number of protein sequences with other organisms) because we wanted to ascertain whether a central organism might be associated with HGT. Next, we added the most central organism from each dataset to the other two sets for a final count of 360 organisms and approximately 1.2 M sequences in each dataset. Organisms in the three datasets are listed in Supplementary Tables S2–S4.

The software package *pClust* was used to cluster the complete proteomes deduced from the genomes selected for the three separate datasets. The largest number of sequences, 1.2 M, grouped into 110,495 non-singleton clusters. Each non-singleton cluster consisted of homologous protein sequences with each sequence representing a unique organism but not the reverse, that is, a cluster could contain more than one sequence for the same organism. The non-singleton cluster results for the three datasets are available online [14].

### 3.2. Heatmap and Essential Genes

We created a heatmap of protein sequence cluster membership together with hierarchical clustering results for each of our datasets to assist in understanding the cluster results. The heatmap for Dataset 1 is shown in Figure 1. Cyan represents membership in a protein cluster while blue indicates absence. The legend of phyla at the top of the figure shows the largest clades represented by colored bands immediately above the heatmap. On the left side of the dendrogram we find Actinobacteria, with distinct blocks of clusters indicated by cyan (for example, A ad B in Figure 1). Bacteriodetes (black with yellow lines) also has blocks of protein clusters (for example, C in Figure 1). Supplementary Figures S1 and S2 show heatmaps for the other two datasets. A dense cyan bar is observed from the left side of the heatmap to the right side in all three datasets (designated by yellow arrows), prompting analysis of the clusters with many shared protein sequences. We found 49 proteins were shared by at least 99% of the bacteria represented by the 360 proteomes. The same 49 proteins were found in 99% of the bacteria in Dataset 1 and Dataset 2 as well. Based on this prevalence, we deduced these 49 to be essential proteins. In addition, we found that 122 proteins were shared by >94% of the organisms in Dataset 1. There were variations in the two additional datasets as might be expected given their different compositions, but these variations were slight. Supplementary Tables S5 and S6 list the proteins that are shared between ≥99% and >94% of organisms, respectively, for Dataset 1.

### 3.3. Tree of Phyla

The tree of life was constructed using physical observations until the end of the last century when Woese proposed the use of molecular methods [32]. Woese used a single gene (small subunit rRNA) to build a tree of life with three domains, bacteria, archaea, and eukarya. Later the use of a single gene was challenged by several researchers [8,33] who proposed replacing this method by phylogenomic methods which use more than a single gene/protein, typically concatenating the sequences.

Two recent studies presented bacterial trees based on concatenated protein sequences. The Hug dataset used 16 different ribosomal protein sequences while the Parks dataset employed 120 ubiquitous single-copy proteins [8,33]; however, these two phylogenomics approaches relied on ad hoc means for selecting the proteins used to build their trees. We propose a systematic approach that uses highly conserved, essential proteins shared across phyla identified by clustering proteomes deduced from complete genomes to create phylogenetic trees. While clustering was performed on protein sequences, we used DNA sequences to construct our high-resolution tree of bacterial phyla.

### 3.4. Network of Organisms and Horizontal Gene Transfer

**Figure 2:**
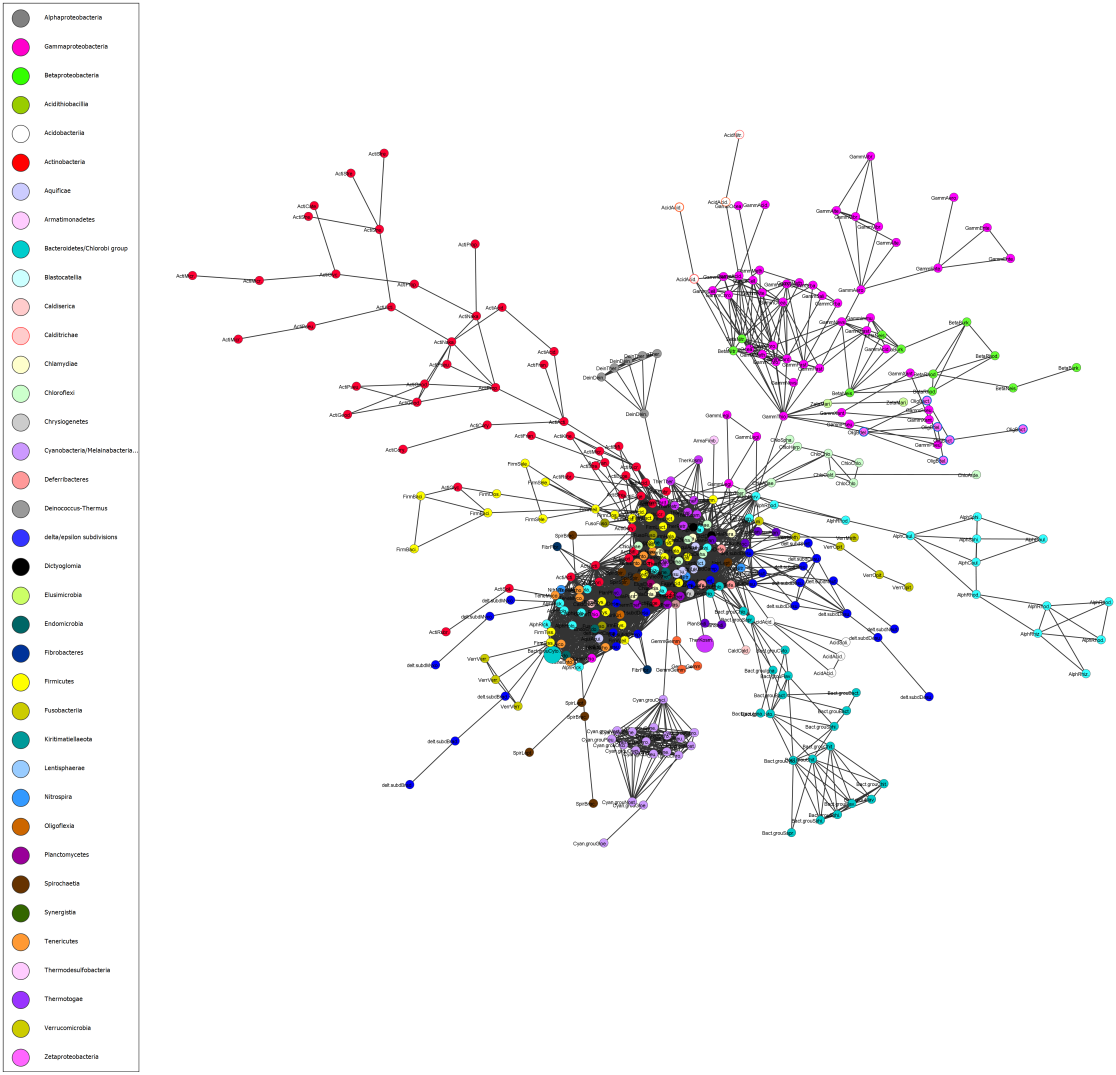
A network of organisms assembled using homologous clusters obtained from the proteomes deduced from 360 complete bacterial genomes and retaining only the top 20% of the links between organisms (shared protein sequences). Phyla are assigned arbitrary colors. Nodes/Organisms are labeled using the first four letters of their particular phylum followed by their order. The network is created from all proteins shared by non-singleton (two or more sequences from different organisms) clusters. Therefore, it reflects the relationships between organisms both vertically and horizontally. In the network, organisms belonging to the same phylum often group together, e.g., Chloroflexi (seafoam green), Actinobacteria (red), Bacteroidetes/Chlorobi (light blue), and Cyanobacteria (light purple). The central section of the network has many organisms that are strongly linked to each other indicating that they share many homologous protein sequences. The Firmicutes (yellow) have a grouping that extends from the middle of the network, but are also distributed throughout this section. The nodes with larger circles indicate organisms isolated from their respective phyla; as discussed in the text, these organisms have numerous horizontally-transferred genes.

We created a network of bacterial organisms by computing distances between organisms. Figure 2 presents the network of organisms assembled using the homologous clusters obtained for the 360 organisms in Dataset 1, retaining only the top 20% of links between organisms (see Section 2). Supplementary Figures S3 shows a dendrogram of the tree given in Figure 2. Of particular note is that our tree has only three phyla with isolated organisms.

In Figure 2, organisms belonging to the same phylum often group together, e.g., Chloroflexi (seafoam green), Actinobacteria (red), Bacteroidetes/Chlorobi (light blue), and Cyanobacteria (light purple). Cyanobacteria are autotrophs and the only prokaryote capable of oxygenic photosynthesis [34], which has allowed them to live on earth over three million years without a major exchange of genes, explaining their isolated grouping. In contrast, the Firmicutes (yellow) have a grouping that extends from the middle of the network, but are also distributed throughout this section. In fact, the central section of the network has many organisms that are strongly linked to each other indicating that they share many homologous protein sequences and potentially instances of HGT. HGT from one phylum to another can occur quickly. A recent study showed that antibiotic resistance plasmid RP4 is likely to be transferred to bacteria in 15 different phyla within 75 days [35]. Analyzing the network may provide information about transfers of this nature. In particular, occasionally one or two members of a phylum cluster separately from the rest. These standouts have more links with members of other phyla than with members of their own phylum and, thus, are strong candidates for HGT. Two such examples are *Cardinium endosymbiont of Sogatella furcifera* (*Cardinium* cSfur) of the phylum Bacteroidetes (large light blue circle), which is actually the most central organism in Dataset 1, and *Mesotoga infera* of the phylum Thermotogae (large purple circle) (Figure 2; note that 80% of the links between organisms showing shared proteins have been pruned from the tree to allow a clear picture of the strongest links). In fact, Zheng et al. [36] demonstrate that *Cardinium* cSfur has undergone a high proportion of horizontal gene transfer and provide a list of 40 candidate genes. There are four categories of candidate horizontally-transferred genes in *Cardinium* cSfur: biotin synthesis genes, glycolysis-related genes, transposase-encoding genes, and other non-transposase encoding genes.

Generally, the mechanisms for inferring HGT events can be divided into two groups: parametric and phylogenetic methods [37]. Parametric approaches detect genes transferred via HGT based on compositional characteristics, such as GC content, codon usage, and the oli-, di,- and tetra-nucleotide frequencies of a genome. Phylogenetic methods seek to identify genes whose relationships contrast sharply with those inferred from other genes in the genome. In this work, first we apply the parametric approach to identify the likely HGT events, then we create the gene tree to infer gene donors.

**Figure 3:**
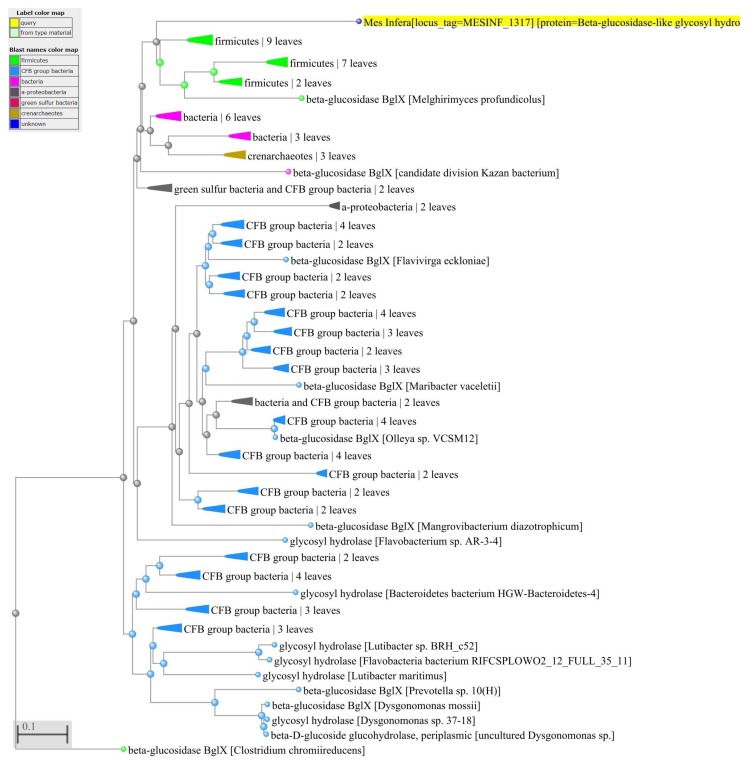
Distance tree for genes horizontally transferred to *M. infera*. The distance tree for MESINF_1317 was searched against the NCBI NR database using BLASTp. It indicates that MESINF_1317 is a *Beta glucosidase-like glycosyl hydrolase* protein potentially transferred from an organism of the order Bacillales, phylum Firmicutes. For a greater portion of the horizontally-transferred genes (55%), protein function is unknown, and inferring origin of the gene can be helpful in predicting protein function.

**Figure 4:**
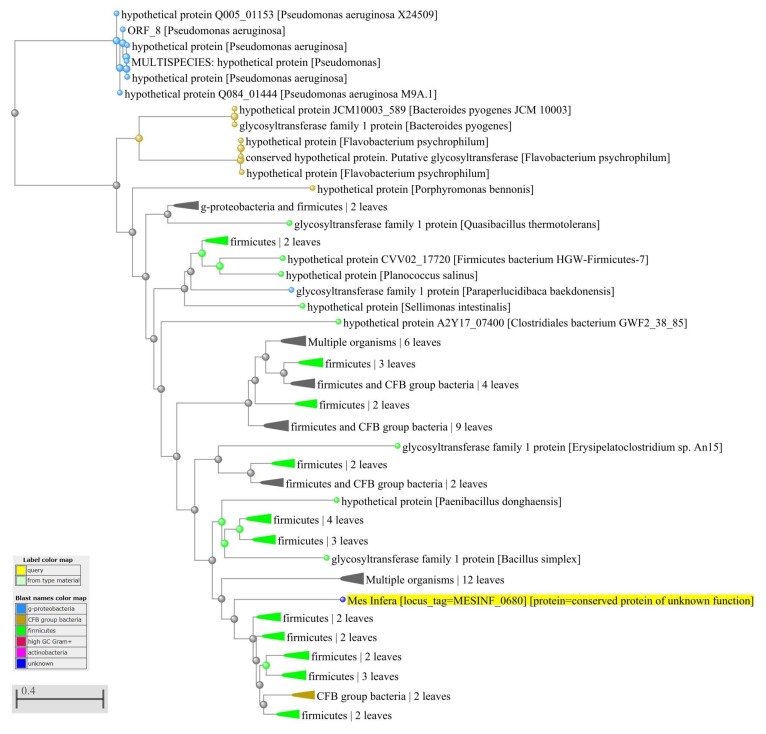
Distance tree for genes horizontally transferred to *M. infera*. The distance tree for MESINF_0680 indicates that it may be a glycosyltransferase family protein transferred from *Paenibacillus* sp. FJAT-27812.

To examine HGT in *M. infera* we used the method developed by Garcia-Vallve [10] (see Section 2). Of the 2823 genes in *M. infera*, 79 appear to have been acquired from other phyla. Supplementary Table S7 lists the 79 possible horizontally-transferred genes, their length, and their G + C content. For a portion of the horizontally-transferred genes (45%), protein function is known and is much the same as the protein function from the donor bacterium. For example, Figure 3 shows the distance tree for MESINF_1317 that was searched against the NCBI NR database using BLASTp [28]. We used BLAST to visualize the distance tree [38] in Figure 3 and Figure 4. It indicates that MESINF_1317 is a Beta glucosidase-like glycosyl hydrolase protein potentially transferred from an organism of the order Bacillales, phylum Firmicutes. For a greater portion of the horizontally-transferred genes (55%), protein function is unknown, and inferring origin of the gene can be helpful in predicting protein function. For example, Figure 4 shows the distance tree for MESINF_0680 indicating that it may be a glycosyltransferase family protein transferred from *Paenibacillus* sp. FJAT-27812. Interestingly, exploring the protein sequence of MESINF_0680 using gene ontology (GO [29]) validated our finding for the protein, which is a putative colanic acid biosynthesis glycosyl transferase WcaC subfamily, glycosyltransferase family. Supplementary File S1 lists the likely protein functions for the portion of the horizontally-transferred genes of *M. infera* with unknown functions found by analyzing the probable origin of donor genes.

**Figure 5:**
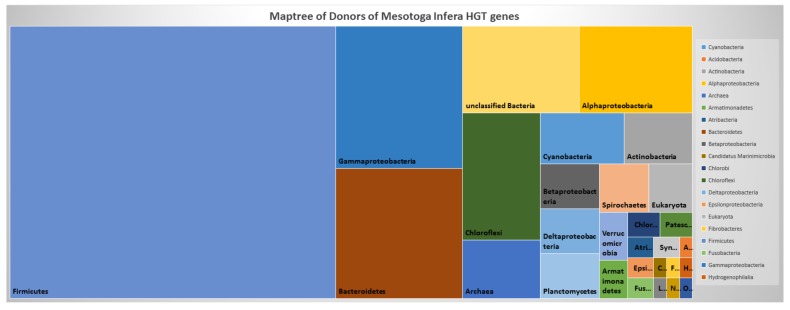
A maptree of the distribution of donor phyla for the horizontally-transferred genes of *M. infera*. The genes are mainly from the orders Bacillales and Clostridiales in the phylum Firmicutes, followed by Gammaproteobacteria, Bacteroidetes, Chloroflexi, Alphaproteobacteria, and Archaea.

Figure 5 displays a maptree of the distribution of donor phyla for the horizontally-transferred genes of *M. infera*. Notably, the genes are mainly from the orders Bacillales and Clostridiales in the phylum Firmicutes, followed by Gammaproteobacteria, Bacteroidetes, Chloroflexi, Alphaproteobacteria, and Archaea. It is of note that some studies based on 16S rRNA have suggested there is a close relationship between Archaea and Firmicutes with Thermotogae [39]. As we have demonstrated, our network of organisms can be used to detect organisms that have participated in HGT. Moreover, once the horizontally-transferred genes have been identified, protein function can be inferred if the function is known for the protein from the organism most closely associated with HGT.

## 4. Discussion

### 4.1. Heatmap and Essential Genes

Many of the 49 essential proteins used to construct our tree are those involved in translation, but not all (25 of them are not). The point of our study was to find genes that were conserved across the breadth of bacterial phyla so they could be used in a study such as this. Other studies have solely used ribosomal proteins, e.g., [8], or ad hoc methods of identifying the selected genes or proteins for inclusion. We identified the 49 proteins in a systematic manner. In some studies, researchers analyzed a much smaller set of related taxa (i.e., more closely related) and, thus, could use a more diverse set of proteins. However, we were specifically searching for a set of proteins that can be used for bacteria in all recognized phyla. It is not surprising to find ribosomal proteins to be widely conserved, as the ribosome, composed of a small and large subunit, is the basic unit for protein translation, a task common to all organisms [40,41,42,43,44,45]. The 30S ribosomal subunit is composed of 24 small proteins and one molecule of ribosomal RNA (16S rRNA) [40]. The 50S large subunit contains two rRNA molecules (23S and 5S rRNA) and 33 proteins [41]. The proteins shared by 94% of the bacteria in our study are a subset of those predicted as part of the minimal gene content of the last universal common ancestor in previous studies [46,47] and are in agreement with other studies on the minimal gene set that were published between 1995 and 2016 [48,49,50]. Of the 122 proteins shared by >94% of the organisms in our dataset, 42 were 30S or 50S ribosomal proteins. Given that ribosomal proteins are among the most highly conserved of all proteins, these results are not surprising.

### 4.2. Tree of Phyla

**Figure 6:**
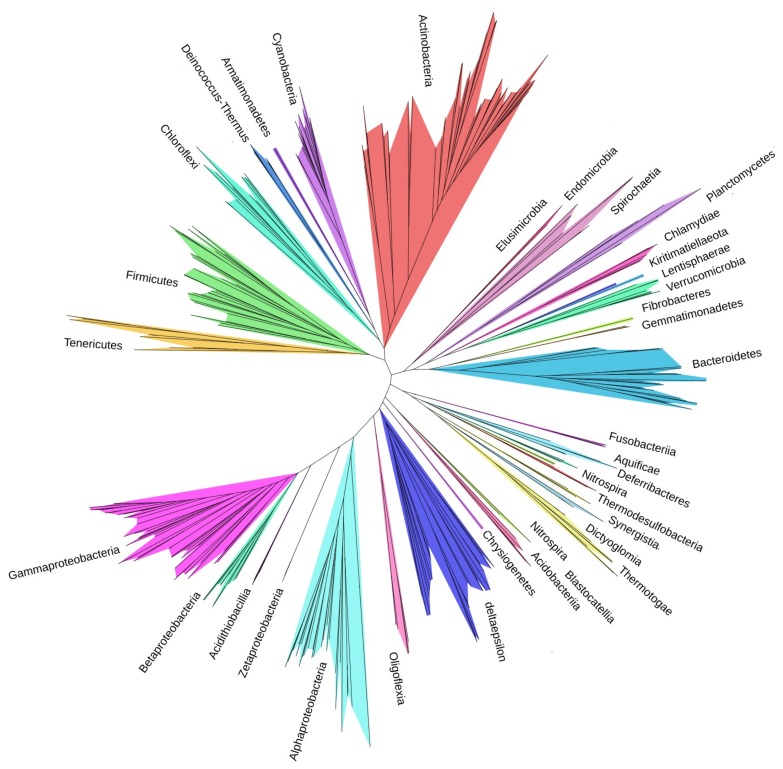
A high-resolution tree of bacterial phyla created from 49 proteins shared by ≥99% of the 360 bacterial organisms in our study. Each phylum is specified using a distinct color. The major bacterial lineages based on orders are Actinobacteria and Gammaproteobacteria, followed by Firmicutes, Alphaproteobacteria, and Bacteroidetes. Proteobacteria classes, excluding the delta/epsilon subdivision, appear on the same branch. A dendrogram of the tree including the complete organism names and phyla is available in Supplementary Figure S5.

Our tree (Figure 6) shares similarities to those presented by other researchers such as Hug [8] and Schulz [9]. For example, the distribution of phyla in the three trees share some similarity. Almost all Proteobacteria classes are grouped together on the same branch, and all three have Firmicutes and Tenericutes closely clustered together on the same branch. Twenty-two of the 49 proteins used to build our tree (Supplementary Table S6) are ribosomal proteins (S2, S3, S4, S5, S7, S8, S9, S10, S15, L1, L2, L3, L5, L6, L7, L11, L13, L14, L15, L16, L18, L19), 11 of which were in the set of 16 proteins used in the Hug dataset. However, in our case the ribosomal proteins are augmented by other highly conserved proteins such as Elongation Factor Tu (Ef-Tu) [51] which has a high G+C ratio that limits the sequence from changing easily.

Our analysis demonstrates that the major bacterial lineages based on orders are Actinobacteria and Gammaproteobacteria, followed by Firmicutes, Alphaproteobacteria, and Bacteroidetes. Proteobacteria classes appear on the same branch. Supplementary Figure S3 shows a dendrogram of the tree given in Figure 6. Of particular note is that our tree has only three groups with isolated representatives while the remainder are clustered together into discrete branches (see Firmicutes, Spirochaetia, and delta/epsilon Proteobacteria in Figure 6). This implies that more than 99% of the major phyla lineages are grouped together. The same is not true for previous trees [8,9]. Our tree is neither biased by overrepresentation of certain clades, nor affected by lack of diversity in the dataset. In addition, construction of the tree by concatenating highly conserved proteins shared by at least 99% of the organisms has resulted in a robust phylogeny.

When we decrease the percentage of genes shared by organisms from 99% to 94% of the organisms in this study, the number of genes in common increases significantly. We speculate that a long time ago bacteria shared more genes, but with the passage of time, the numbers decreased as bacteria found specialized niches and genes evolved to make them more fit for these niches. The occurrence of HGT, which we know is responsible for genes that help bacteria survive, has contributed to the complex relationship among bacteria today.

### 4.3. Network of Organisms and Horizontal Gene Transfer

**Figure 7:**
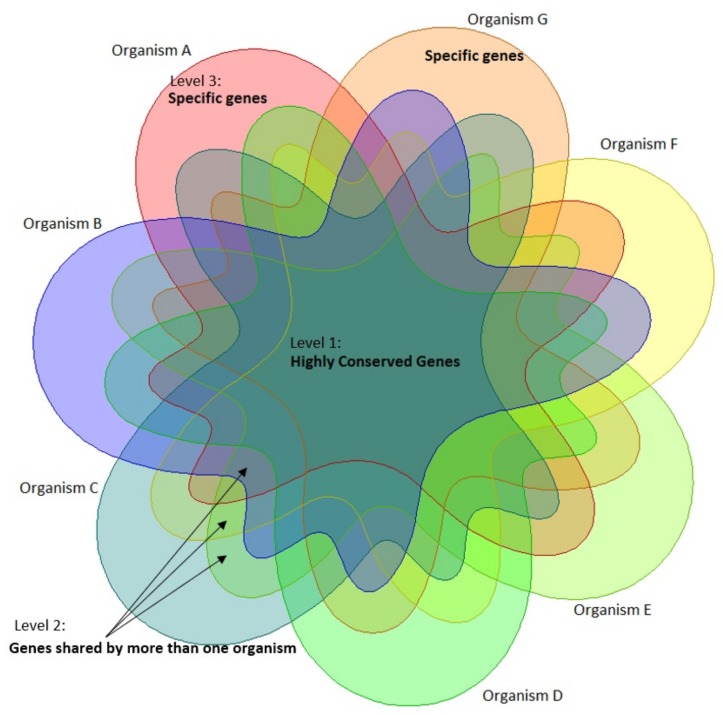
A Venn diagram depicting how genes from multiple organisms cluster: Genes that are shared by all organisms, genes that are shared by more than one organism, and genes that are specific to one organism.

The tree of life reflects the current knowledge of the evolutionary relationships between organisms with the passage of time. However, viewed from a more dynamic perspective the genomes of bacterial organisms are actively changing in terms of their size and content because of such phenomena as gene loss, duplication of genes within genomes, and acquisition of genes from foreign sources by means of HGT. HGT in bacteria occurs by means of three mechanisms: conjugation by which DNA is transferred directly between bacteria, transformation by which bacteria absorb free DNA from the environment, and transduction by which bacteriophages (bacterial viruses) transfer DNA to bacteria [52]. There are three levels of gene sharing by bacteria within a phylum (Figure 7). The first level consists of genes that are highly conserved and shared by all bacterial organisms. The second level includes genes that are present in more than one organism. At this level the transfer of genes occurs frequently as organisms are closely related [53]. The third level has genes that are specific to a particular organism. The transfer of genes from other phyla might happen at this level. When clustered, third-level genes are either present in the smaller clusters or else they are not homologous to any other gene and form singleton clusters. Singletons might be the result of gene transfer by phages [52]. The network we created in this work is based on shared proteins in the three levels (Figure 2). Our network addresses the relationships between organisms using genes acquired both vertically and horizontally. Such a network can assist in the discovery and understanding of complex relationships between organisms such as HGT. Furthermore, we used centrality measures to enhance our analysis. When we added the most central organism from each of the three datasets to the other datasets, one organism was the most central organism. Interestingly, this organism *Cardinium* cSfur was found to include a large number of horizontally-transferred genes.

## 5. Conclusions

In this paper we reported the need to use orders rather than phyla to reflect the diversity of organisms within phyla. Importantly, we introduced a systematic approach for choosing protein sequences deduced from essential genes for use in creating an accurate tree of life using homologous clusters of protein sequences shared by ≥99% organisms and have illustrated this approach for 360 different bacterial organisms. While our interest was restricted to bacteria, we determined that 47 of the 49 genes used to create our tree are shared by representatives of both archaea and eukarya (Supplementary Table S8). Finally, we have shown how homologous protein clusters can be used to generate a network of bacterial organisms and have confirmed that such a network can be used to identify bacterial genomes containing many horizontally-transferred genes.

## Supplementary Materials

The following are available online at https://www.mdpi.com/2076-2607/8/2/312/s1. Supplementary Table S1. A list of orders and their availability at NCBI. This table contains a list of recognized phyla, their classes, and their orders at the initiation of the study (August 2019). Also, the number of orders available at NCBI are presented. Supplementary Table S2–S4. Accession numbers, subgroups, groups, and orders for the genomes of the three datasets. Supplementary Table S5. Lists of shared proteins >94% of the 360 bacterial organisms in Dataset1. Supplementary Table S6. Lists of shared proteins 99% of the 360 bacterial organisms in Dataset1. Supplementary Table S7. List of the proteins shared by 99% of bacteria and present in representatives of both archaea and eukarya. Supplementary Table S8. List of 79 genes that are probably horizontally transferred from other phyla to *M. infera*. The length of the genes and their G + C content are also presented. Supplementary Figure S1. Heatmap of the cluster results for the first additional dataset. Supplementary Figure S2. Heatmap of the cluster results for the second additional dataset. Supplementary Figure S3. Dendrogram of the tree of bacterial phyla presented in Figure 6 with the same colors used to represent phyla. Tree labels are complete names of organisms and their phyla. Supplementary Figure S4. Network of organisms for the Dataset2. Supplementary Figure S5. Network of organisms for Dataset3. Supplementary File S1. List of likely protein functions for the portion of the horizontally-transferred genes of *M. infera* with unknown functions found by analyzing the probable origin of donor genes.

## Abbreviations

The following abbreviations are used in this manuscript:

HGT	Horizontal Gene Transfer
